# Activity of antifilarial drugs on microfilaremia in the treatment of loiasis: a systematic review

**DOI:** 10.1186/s13071-025-07189-w

**Published:** 2025-12-10

**Authors:** Pia Michelitsch, Lars Matthies, Tamara Nordmann, Rella Zoleko Manego, Michael Ramharter

**Affiliations:** 1 Center for Tropical Medicine, Department of Medicine, Bernhard Nocht Institute for Tropical Medicine and I, University Medical Center Hamburg-Eppendorf, Hamburg, Germany; 2 German Centre for Infection Research (DZIF), Partner Site Hamburg-Luebeck-Borstel, Hamburg, Germany; 3 Centre de Recherches Médicale de Lambaréné (CERMEL), Lambaréné, Gabon

**Keywords:** Anthelmintic treatments, *Loa loa*, Loiasis, Microfilaremia, Systematic review, Tropical diseases

## Abstract

**Background:**

Loiasis, caused by the nematode/filaria *Loa loa*, presents a major health burden in Central and West Africa. Despite the growing recognition of loiasis’ medical significance, current antifilarial drugs remain inadequate in terms of efficacy and safety, particularly for individuals with hypermicrofilaremia. This systematic review aims to evaluate the efficacy of antifilarial treatment regimens for reducing *L. loa* microfilaremia and provide guidance on treatment strategies.

**Methods:**

A systematic review was conducted to evaluate the efficacy of antifilarial treatment regimens on reducing *L. loa* microfilaremia. Data on the percentage reduction of microfilaremia from baseline to nadir were extracted for each treatment regimen.

**Results:**

A total of 27 studies were included in the review, with treatment regimens involving albendazole (ALB), mebendazole (MBZ), ivermectin (IVM), diethylcarbamazine (DEC), levamisole, imatinib, and moxidectin, among others. ALB and MBZ showed dose- and duration-dependent efficacy, with extended treatment leading to up to a 98–100% microfilaremia reduction. IVM showed a dose-dependent effect, with single doses of 200–400 µg/kg reducing microfilaremia by 88–92%. DEC exhibited high efficacy, achieving up to a 100% microfilaremia reduction.

**Conclusions:**

Antifilarial drug efficacy against *L. loa* microfilaremia varies by dosage and treatment duration, with IVM and DEC demonstrating rapid, high efficacy but presenting safety concerns for hypermicrofilaremic individuals. ALB and MBZ show efficacy with extended treatment but are slower acting. Further research is needed to optimize treatment regimens and assess clinical outcomes beyond microfilaremia reduction.

**Graphical Abstract:**

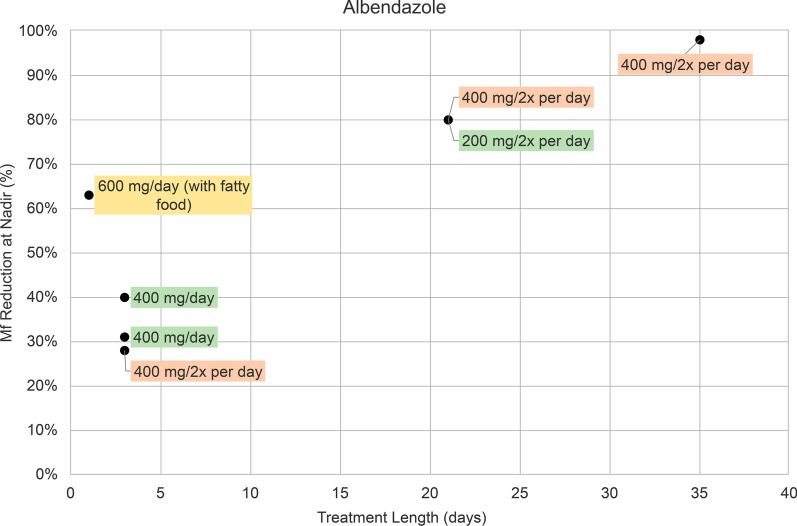

**Supplementary Information:**

The online version contains supplementary material available at 10.1186/s13071-025-07189-w.

## Background

Loiasis, commonly referred to as African eye worm, is a disease caused by the nematode *Loa loa* and affects millions of individuals living in endemic forest and savanna regions of Central and West Africa [1, 2]. Deer flies (*Chrysops* spp.), which breed in rural rainforest regions, serve as vectors and may transmit *L. loa* to human hosts, where the infective larvae develop into adult worms [1, 3]. Patients may subsequently suffer from occult infection, with only adult worms present, or may feature microfilariae in peripheral blood. *L. loa* may lead to exceptionally high microfilaremia levels above 100,000/ml peripheral blood [3]. Infection can also cause clinical symptoms, including pruritus, peripheral transient angioedemas known as Calabar swellings, or eye worm migration [4]. Moreover, the clinical presentation may encompass both common, nonspecific symptoms and rare, yet potentially life-threatening complications [4].

Loiasis is one of the most common reasons for medical consultation in some areas [2]. Recently, two landmark studies have changed our understanding of the true burden of loiasis. Patients with hypermicrofilaremia have been demonstrated to suffer significant excess mortality in a retrospective population-based cohort study from Cameroon [5]. Similarly, a cross-sectional survey study in Gabon established the disability-adjusted life years lost due to loiasis, indicating that its overall burden is comparable to other leading neglected tropical diseases, such as schistosomiasis, in this setting [6].

Treatment of loiasis may not only lead to improvement of clinical signs of the disease and eventual cure from infection in individual patients, but may also prove important for the potential control of transmission in mass drug administration programs. However, currently, none of the antifilarial drugs used to treat loiasis are sufficiently safe, efficacious, and easy to administer to be used on a large scale.

In light of the now-recognized medical importance of loiasis, the lack of effective therapeutic interventions is untenable. Each available therapeutic drug for loiasis has distinct limitations, hindering its use for individual patient care and the control of this microfilarial disease. Importantly, the available evidence for the efficacy, safety, and tolerability of antifilarial drugs for loiasis has not been systematically scrutinized for evidence-based conclusions. Thus, an overview of all currently available evidence on the efficacy of loiasis treatment regimens in reducing microfilarial density (MFD) is of major importance in filling this gap.

This systematic review evaluates the efficacy of various established treatment regimens for reducing and ultimately clearing microfilaremia in patients with *L. loa* infection. In addition, analysis of the dose–response characterization for respective antifilarial drugs was attempted when sufficient data were available to provide guidance on optimal treatment doses and durations.

## Methods

### Search strategy

We searched for articles published until 31 July 2024 on the PubMed database using the terms “Loa OR loiasis”.

### Inclusion criteria

As per our protocol (PROSPERO CRD42024558036), we included studies with at least ten human participants for the treatment of loiasis. The threshold of ten was employed to exclude small case series, which are prone to important selection bias. The included studies reported on the treatment of returning travelers or residents of endemic regions in Central and West Africa. Inclusion was limited to studies with MFD data available at baseline and nadir or studies reporting the change in MFD from baseline to nadir. Clinical trials, including but not limited to randomized controlled trials, prospective cohorts, retrospective cohorts, and case series were considered. Only articles published in English, French, German, and Spanish were eligible for inclusion.

### Exclusion criteria

Studies reporting outcomes on adverse events and serious adverse events (SAEs) but not reporting the observed treatment effect on microfilaremia, were not considered. Studies with no microfilarial values as an outcome or insufficient data were excluded. Nonhuman trials, summaries, and reviews on the topic of *L. loa* or loiasis were also excluded.

### Risk of bias assessment

Risk of bias of included studies was assessed using three tools: the revised risk of bias in randomized trials (RoB2) tool [7], the risk of bias in nonrandomized studies of interventions (ROBINS-I) tool [8], and the methodological index for nonrandomized studies (MINORS) [9]. We visualized the risk-of-bias assessment results through generating risk-of-bias plots using the R *robvis* package by McGuinness et al. [10].

For randomized trials, the RoB2 tool evaluated five domains as follows: randomization process, deviations from intended intervention, missing outcome data, outcome measurement, and selection of the reported result. One randomized crossover trial was assessed on the additional RoB2 domain of bias arising from period and carryover effects.

Nonrandomized studies comparing at least two study arms were assessed using the ROBINS-I tool, which covers the following seven domains: confounding, participant selection, intervention classification, deviations from intended interventions, missing data, outcome measurement, and selection of reported results.

Noncomparative studies were scored using the MINORS criteria across the following eight domains: a clearly stated aim, inclusion of consecutive patients, prospective data collection, endpoints appropriate to the aim of the study, unbiased assessment of the study endpoint, follow-up period appropriate to the aim of the study, loss to follow-up less than 5%, and prospective calculation of the study size.

### Data extraction

One author independently identified and screened the studies according to the inclusion and exclusion criteria. A second author reviewed all identified studies. Two authors independently extracted data, which included demographics of participants (age and sex), treatment (drug name, dose, length of treatment and number of treated subjects), laboratory (MFD [mf/ml]), and clinical (subjects with eye worm, Calabar swelling and other clinical symptoms presumptive of loiasis) parameters at baseline and nadir (lowest reported level of microfilaremia) and after treatment.

The treatment effect was derived from the percentage of change in the median, geometric mean, or arithmetic mean (depending on reported measures and the distribution of data) of MFD from baseline to nadir. Each drug regimen from the selected eligible studies was included in a table with the length of treatment, percent reduction of microfilaremia at nadir and endpoint, and time to nadir and endpoint. Graphs illustrating the percent reduction of microfilaremia in relation to treatment length were derived from the data of the doses for each study drug.

## Results

### Search result

**Fig. 1:**
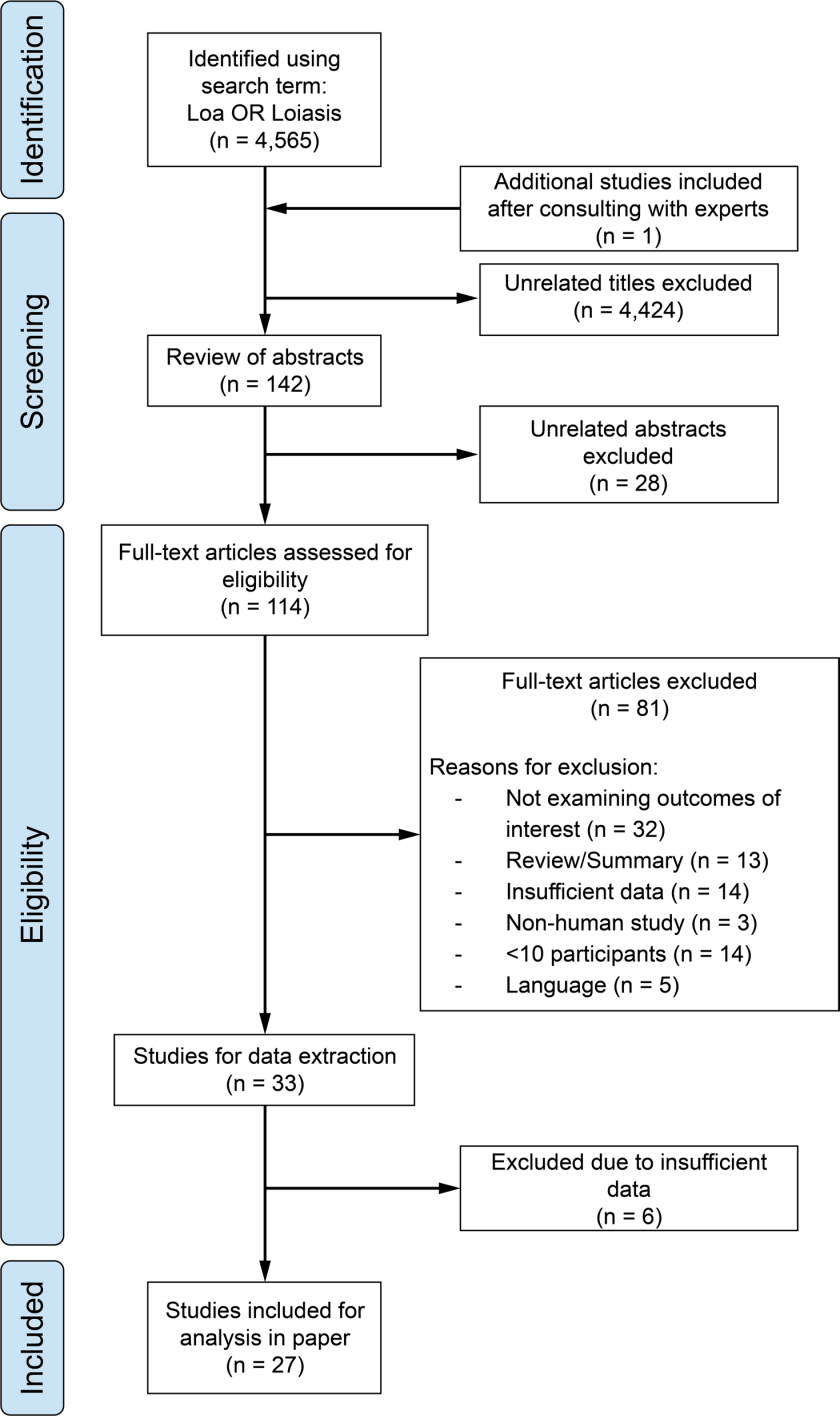
Preferred Reporting Items for Systematic Reviews and Meta-Analyses (PRISMA) flowchart summarizing the literature search results

The flow of identification and selection of studies is shown in Fig. 1. Our literature search identified 4565 records. A total of 114 studies were selected for full-text evaluation. Of these, 81 articles were excluded for the following reasons: they did not examine the outcomes of interest (*n* = 32), were reviews or summaries (*n* = 13), lacked sufficient data (*n* = 14), involved nonhuman subjects (*n* = 3), included fewer than ten participants (*n* = 14), or were published in languages other than English, French, German, or Spanish (*n* = 5). An additional eligible study, not captured in the initial PubMed search, was identified through a supplementary search of ClinicalTrials.gov and included in the final analysis. Ultimately, we included 27 studies that met the eligibility criteria and provided sufficient data [11–37].

### Study characteristics

In total, 27 studies contributed data on *L. loa* MFD measured either at baseline and nadir or as the percentage of change of MFD from baseline to nadir (Table 1). We utilized the MFD data as reported in the original studies, whether presented as medians, geometric means, or arithmetic means. No transformations or adjustments were applied to the data for this systematic review. Few studies included in the analysis reported on clinical treatment outcomes, and only a proportion of studies had data on indirect markers of treatment success including eosinophilia.

**Table 1:** Study characteristics

Author, date [reference]	Country	Sex, age range (years)	Treatment	Dose	Treatment length	Mf reduction from baseline to nadir (%)	Time to nadir
Burchard and Kern 1987 [11]	Gabon	NA, ≥ 18	MBZ	1 g BID	21 days	46	D15
Campillo et al. 2022 [12]	Congo	M + F, 18–65	Levamisole	2.5 mg/kg/day	1 day	13	D2
			Levamisole	1.5 mg/kg/day	1 day	8	D2
			Levamisole	1 mg/kg/day	1 day	−10	D30
Carme et al. 1991 [13]	Congo	NA, NA	IVM	200 µg/kg	1 day	91	D14
Duke et al. 1961 [14]	Nigeria	NA, NA	DEC	200 mg TID followed by 200 mg/month	20 days + 6 months	80	Mo7
			DEC	200 mg TID	20 days	63	Mo7
Duong et al. 1997 [15]	Gabon	M + F, 17–76	IVM	200 µg/kg/day	1 day	90	Mo10–Mo12
Herrick et al. 2017 [16]	Cameroon	M + F, 20–70	DEC	8 mg/kg/day	1 day	100	D1
			IVM	200 µg/kg/day	1 day	90	D2
Hoegaerden et al. 1987 [17]	Gabon	NA, 10–62	MBZ	100 mg TID (300 mg/day)	28 days	100	D200
			MBZ	100 mg TID (300 mg/day)	45 days	100	D200
			MBZ	500 mg TID (1500 mg/day)	21 days	100	D14, D21, D40, D60
			MBZ	500 mg TID (1500 mg/day)	28 days	100	D100
			MBZ	500 mg/day	45 days	93	D60
Kamgno et al. 2000 [18]	Cameroon	M + F, ≥ 18	IVM	150 µg/kg/day	1 day	82	D30
			IVM	50 µg/kg/day	1 day	72	D15
Kamgno et al. 2002 [19]	Cameroon	NA, ≥ 12	ALB	600 mg/day (+ fatty food)	1 day	63	D300
			MBZ	100 mg BID (fasting state)	3 days	0	D0
Kamgno et al. 2007 [20]	Cameroon	M + F, 15–70	IVM	150 µg/kg/day	1 day	77	D15
			IVM	1.5 mg/day	2 days (with a 15-day interval)	58	D30
			IVM	1.5 mg/day (25 µg/kg for a 60 kg person)	1 day	49	D30
Kamgno et al. 2010 [21]	Cameroon	M + F, 14–80	Chloroquine	600 mg/day	3 days	51	D60
			Quinine	600 mg BID	5 days	31	D60
			Amodiaquine	800 mg/day	3 days	22	D90
			Artesunate	200 mg/day	3 days	14	D90
Kamgno et al. 2016 [22]	Cameroon	M + F, 18–65	ALB	800 mg bimonthly	11 months	62	Mo21
			ALB	800 mg bimonthly	3 months	32	Mo14
Klion et al. 1993 [23]	Benin	M, 25–55	ALB	200 mg BID	21 days	80	Mo6
Kombila et al. 1998 [24]	Gabon	M + F, 7–78	IVM	200 µg/kg/month	6 months	99	Mo6
Martin-Prevel et al. 1993 [25]	Gabon	NA, NA	IVM	400 µg/kg/day	1 day	88	D5–D8
Martin-Prevel et al. 1993 [26]	Gabon	M + F, 20–55	IVM	300 µg/kg/day	1 day	92	D23–D33
			IVM	400 µg/kg/day	1 day		D23–D33
Murgatroyd et al. 1949 [27]	UK	M + F, NA	DEC	2, 4, or 6 mg/kg/day	10, 14, or 21 days	100	unknown
Nana-Djeunga et al. 2020 [28]	Cameroon	M + F, 5–90	IVM (CDTI)	150–200 µg/kg, 1× per year	18 years	99.8	Y18
O'Connell 2022 [29]	Cameroon	M + F, 19–65	Imatinib	400 mg/day	2 days	40	D4
			Imatinib	200 mg/day	1 day	21	D7
			Imatinib	600 mg/day	3 days	17	D7
Ranque et al. 1996 [30]	Cameroon	M + F, 0–87	IVM	200 µg/kg/every 3 months	2 years	92	Y2
Richard-Lenoble et al. 1988 [31]	Gabon	M + F, 13–72	IVM	100 µg/kg/day	5 days	95	D7
			IVM	200 µg/kg/day	1 day	88	D7
			IVM	300 µg/kg/day	7 days	84	D15
			IVM	150 µg/kg/day	6 days	83	D7
			IVM	30 µg/kg/day	3 days	77	D2
			IVM	50 µg/kg/day	4 days	68	D28
			IVM	5 µg/kg/day	1 day	12	D28
			IVM	10 µg/kg/day	2 days	7	D2
Tabi et al. 2004 [32]	Cameroon	M + F, 15–78	PLA/ALB	400 mg/day	3 days	40	D270
			ALB/PLA	400 mg/day	3 days	31	D90
Tsague-Dongmo et al. 2002 [33]	Cameroon	M + F, 10–70	ALB	400 mg BID	3 days	28	D60
Turner et al. 2010 [34]	Cameroon	M + F, 15–50	DOXY + IVM	200 mg/day DOXY + 150–200 µg/kg/day IVM	6 weeks DOXY + single dose IVM at M4	100	Mo12
Twum-Danso 2003 [35]	Cameroon	M + F, < 15 to ≥ 60	IVM (mass treatment)	150–200 µg/kg/day	1 day	98	Mo1
Wafeu et al. 2024 [36]	Cameroon	M, 18–70	IVM	150 µg/kg	1 day	80	D30
			MOX	2 mg	1 day	48	D30
Zoleko-Manego et al. 2023 [37]	Gabon	M + F, IQR 47–78	ALB	400 mg BID	35 days	98	Mo5
			ALB + IVM	400 mg BID ALB + 150 µg/kg/day IVM	21 days ALB + single dose IVM on D23	96	Mo1
			ALB	400 mg BID	21 days	80	Mo1

*ALB* albendazole, *BID* twice a day, *CDTI* community directed treatment with ivermectin, *D* day, *DEC* diethylcarbamazine, *DOXY* doxycycline, *F* female, *IVM* ivermectin, *IQR* interquartile range, *M* male, *Mo* month, *MBZ* mebendazole, *Mf* microfilaremia, *NA* not available, *PLA* placebo, *TID* three times a day, *Y* year

The included studies were published between 1949 and 2024. With the exception of one, all studies were conducted in endemic areas of central and western Africa, specifically Cameroon (*n* = 14), Gabon (*n* = 8), Congo (*n* = 2), Benin (*n* = 1), and Nigeria (*n* = 1) (Table 1). One study was conducted in London, UK, involving participants who had previously resided in loiasis-endemic regions (Table 1). These studies included participants of both sexes and all age groups, including children and adults (0–90 years of age) (Table 1). Among these, 14 were randomized clinical trials, including one randomized crossover trial. In addition, five studies were nonrandomized and compared at least two study arms, while eight studies were noncomparative.

### Albendazole and mebendazole

**Fig. 2:**
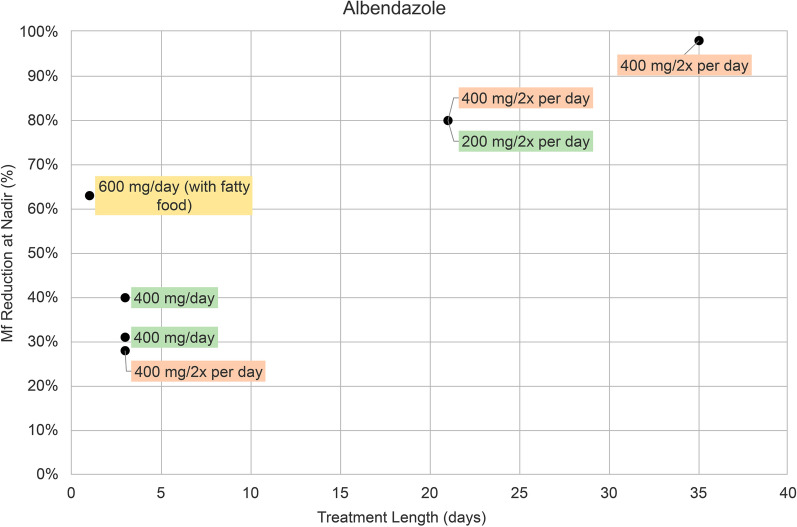
Percentage (%) reduction in microfilaremia from baseline to nadir in seven albendazole treatment groups. Treatment regimens are indicated as follows: 400 mg daily dose (green), 600 mg daily dose (yellow), and 800 mg daily dose (orange). Data points from two treatment groups (800 mg albendazole given bimonthly for 3 and 11 months) were removed. They are included in Table 1. *Mf* microfilaremia

In nine albendazole (ALB) treatment groups there were five different treatment regimens: 200 mg twice per day, 400 mg per day, 600 mg per day, 400 mg twice per day, and 800 mg bimonthly (every 2 months). Shorter-term treatment lengths varied from 1 to 35 days (Fig. 2). ALB 600 mg/per day administered as a single dose resulted in a 63% reduction of microfilaremia at nadir (Fig. 2). ALB given for 3 days resulted in a 28–40% reduction, with a 28% reduction for ALB 400 mg twice per day, and a 31% and 40% reduction for 400 mg/day (Fig. 2). ALB 200 mg twice per day and 400 mg twice per day for 21 days led to an 80% reduction (Fig. 2). ALB 400 mg twice per day for 35 days had a reduction of 98% (Fig. 2). One trial assessed the efficacy of ALB 800 mg administered bimonthly for 3 and 11 months [22], which had reductions of 32% and 62%, respectively (Table 1).

**Fig. 3:**
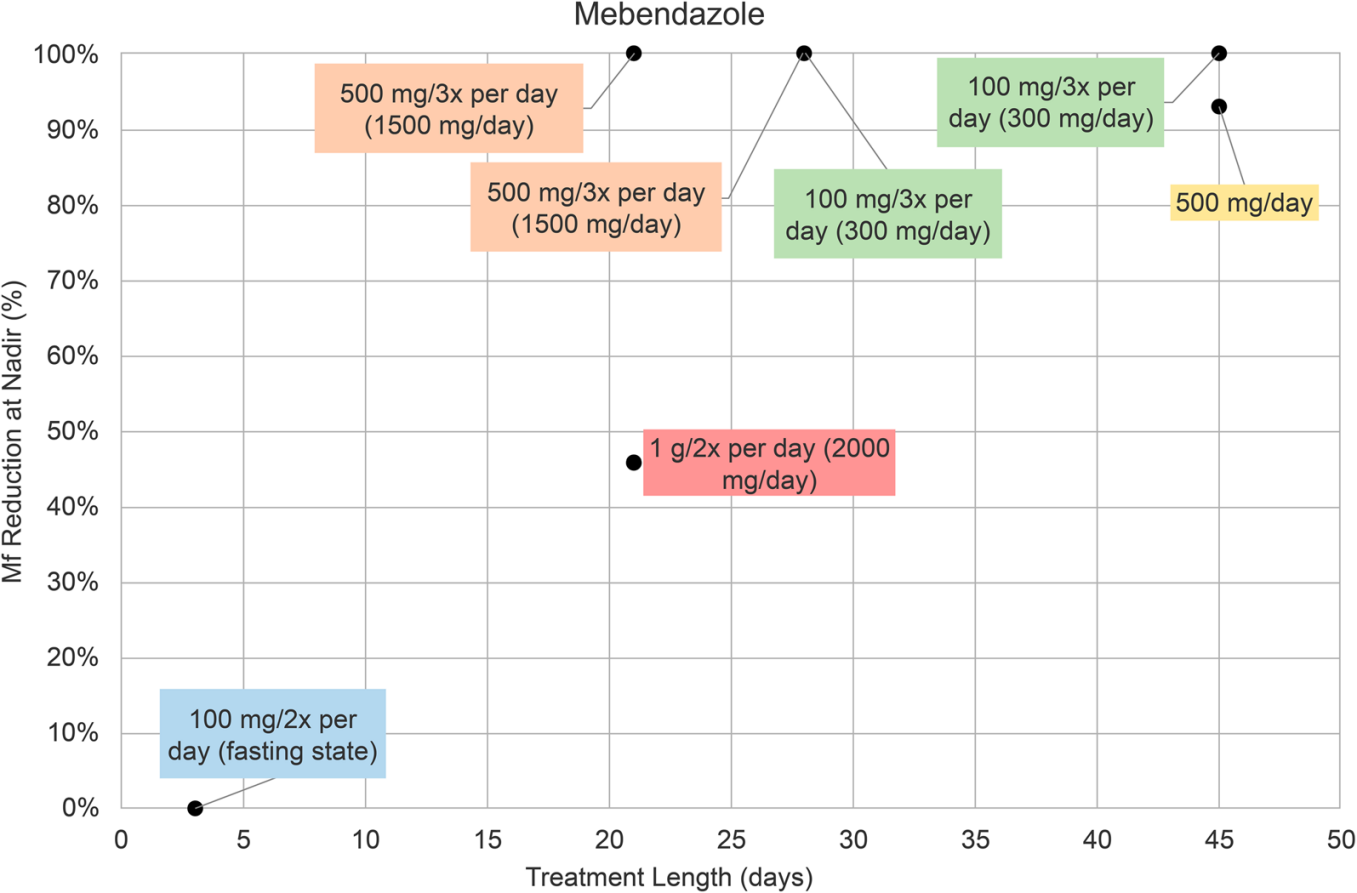
Percentage (%) reduction in microfilaremia from baseline to nadir in seven mebendazole treatment groups. Treatment regimens are indicated as follows: 200 mg daily dose (blue), 300 mg daily dose (green), 500 mg daily dose (yellow), 1500 mg daily dose (orange), and 2000 mg daily dose (red). *Mf* microfilaremia

In seven mebendazole (MBZ) treatment groups, there were five different treatment regimens: 100 mg twice per day, 100 mg three times per day, 500 mg per day, 500 mg three times per day, and 1 g twice per day. MBZ 100 mg twice per day administered for 3 days resulted in a 0% reduction of microfilaremia at nadir (Fig. 3). MBZ 500 mg three times per day and 1 g twice per day were given for 21 days and resulted in a percent reduction of 100% and 46%, respectively (Fig. 3). MBZ 500 mg three times per day and 100 mg three times per day for 28 days both had a microfilaremia reduction of 100% (Fig. 3). MBZ 500 mg/day and 100 mg three times per day for 45 days led to a 93% and 100% reduction, respectively (Fig. 3).

Among ALB studies, time to nadir ranged widely from 1 to 21 months, with most measuring nadir in the first 12 months. The time to nadir reported across the MBZ studies also varied considerably, ranging from 14 to 200 days, with the greatest microfilaremia clearance occurring between 60 and 200 days (Table 1).

### Ivermectin

**Fig. 4:**
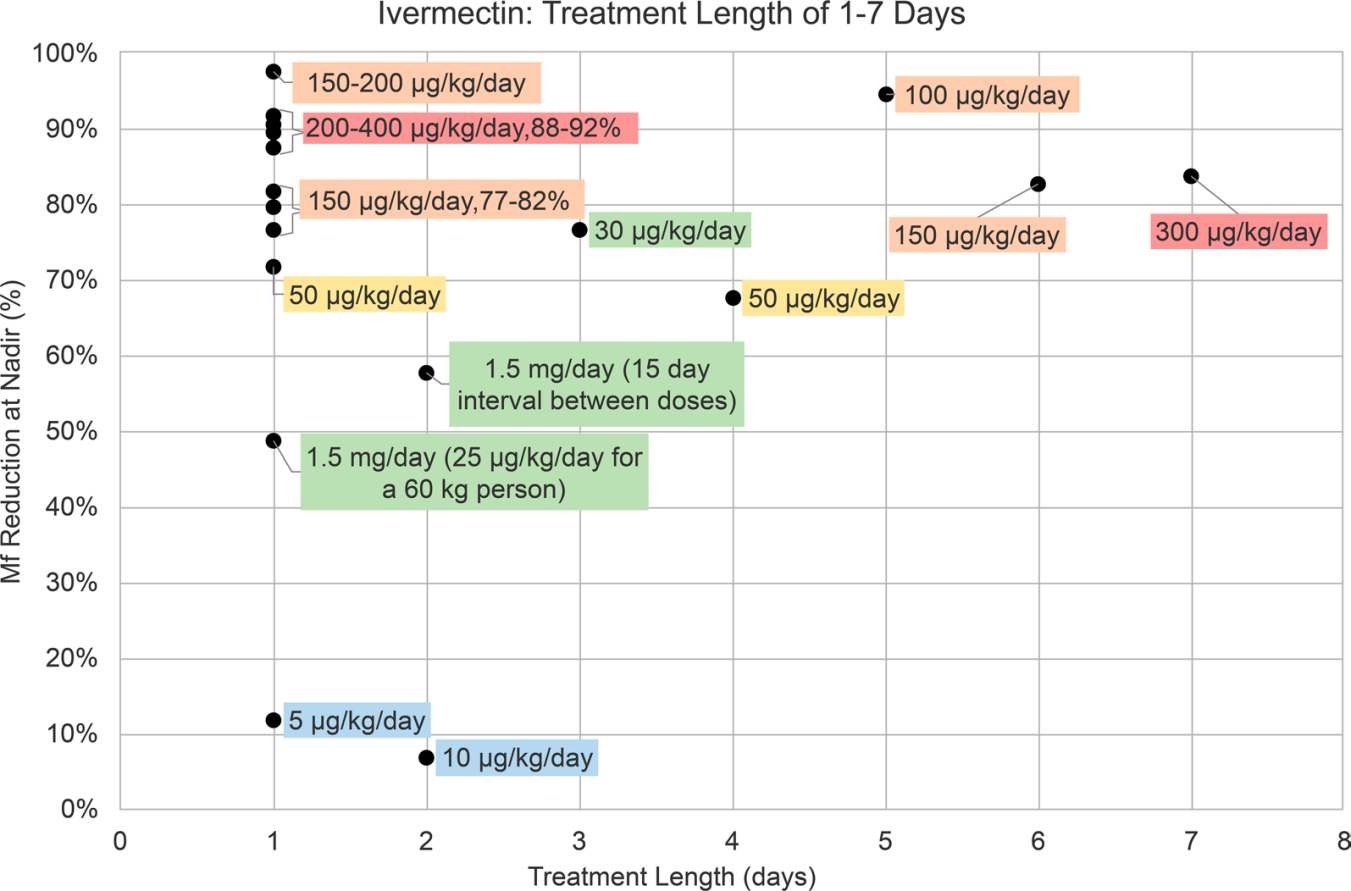
Percentage (%) reduction in microfilaremia from baseline to nadir in 21 ivermectin treatment groups with treatment lengths of 1–7 days. Treatment regimens are indicated as follows: 5–10 µg/kg daily dose (blue), 1.5 mg or 25–30 µg/kg daily dose (green), 50 µg/kg daily dose (yellow), 150–200 µg/kg daily dose (orange), and 200–400 µg/kg daily dose (red). *Mf* microfilaremia

In 21 ivermectin (IVM) treatment groups there were ten different doses: 5, 10, 30, 50, 100, 150, 200, 300, 400 µg/kg/day, and 1.5 mg/day, which were administered for 1–7 days. Among all IVM regimens, IVM 5 µg/kg/day for 1 day and 10 µg/kg/day for 2 days had the lowest percent reduction of 12% and 7%, respectively (Fig. 4). IVM 1.5 mg/day as a single dose had a 49% reduction of microfilaremia, and the same dose administered twice, with a 15-day interval between doses, had a 58% reduction (Fig. 4). IVM 50, 150, 200, 300, and 400 µg/kg/day administered as a single dose had a microfilaremia reduction of 72–92% (Fig. 4). IVM 50 µg/kg/day had the lowest percent reduction of 72%, while doses of 150 µg/kg/day had a 77–82% reduction. Single IVM doses between 200 and 400 µg/kg/day achieved reductions from 88% to 92% (Fig. 4). IVM 30 µg/kg/day for 3 days and 50 µg/kg/day for 4 days had a 77% and 68% reduction, respectively. IVM 100 µg/kg/day for 5 days, 150 µg/kg/day for 6 days, and 300 µg/kg/day for 7 days had percent reductions of 95%, 83%, and 84%, respectively (Fig. 4). Administering a single IVM dose of 150–200 µg/kg in an onchocerciasis mass treatment program resulted in a 98% reduction of *L. loa* microfilaremia (Fig. 4). Nadir MFD measurements generally occurred within the first 30 days after treatment initiation, except for one study where time to nadir was between 10 and 12 months (Table 1).

**Fig. 5:**
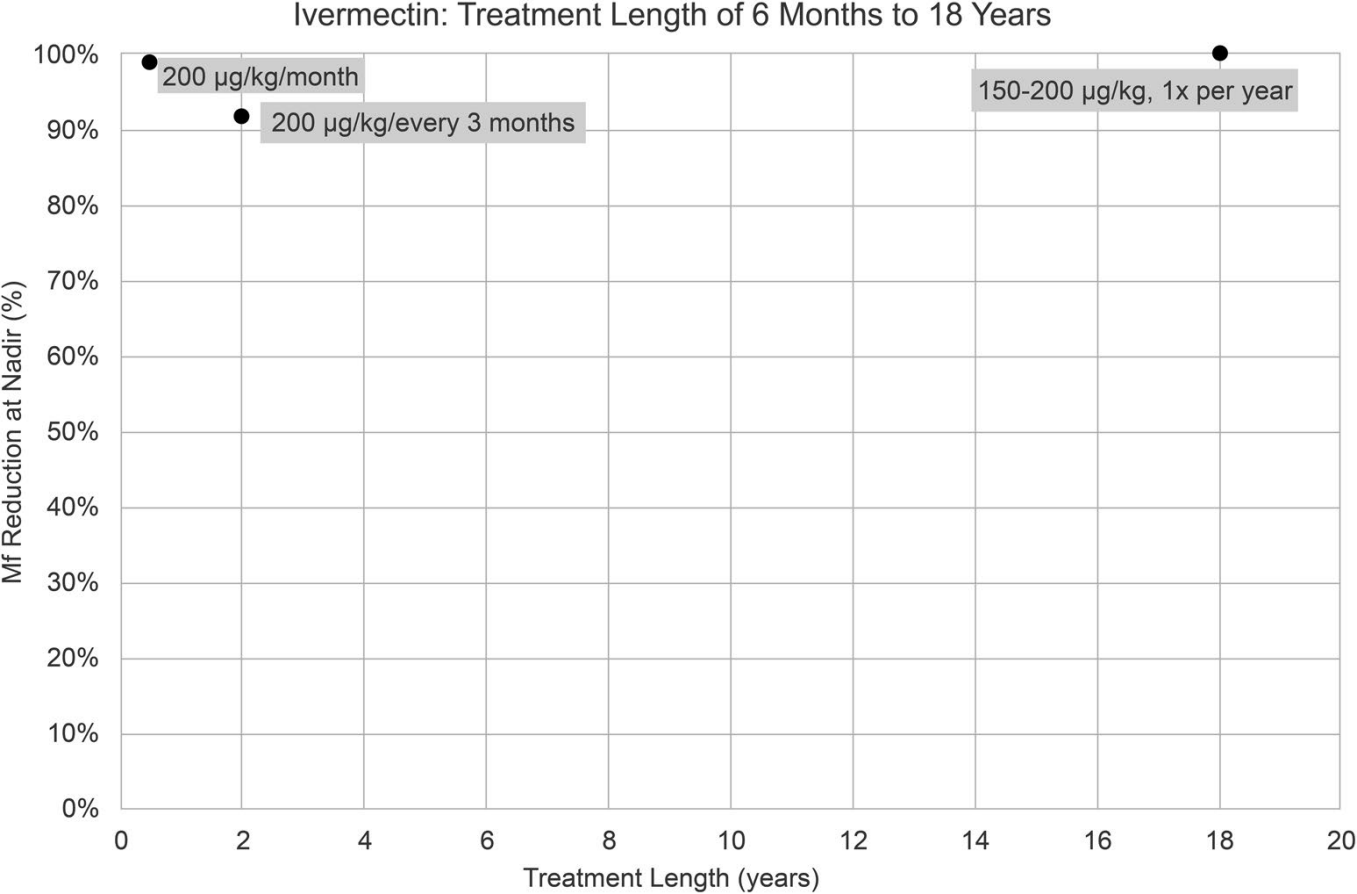
Percentage (%) reduction in microfilaremia from baseline to nadir in three ivermectin treatment groups with treatment lengths of 6 months to 18 years. *Mf* microfilaremia

There were three IVM treatment groups administered for 6 months to 18 years. IVM 200 µg/kg once monthly for 6 months led to a 99% reduction in microfilaremia (Fig. 5). IVM 200 µg/kg administered every 3 months for 24 months (2 years) had a 92% reduction (Fig. 5). Community-directed treatment with IVM for onchocerciasis, administered at 150–200 µg/kg once yearly for 18 years, had a 100% reduction of *L. loa* microfilaremia (Fig. 5). Time to nadir was at the end of the treatment course for each of these studies (Table 1).

### Diethylcarbamazine

A single dose of 8 mg/kg/day diethylcarbamazine (DEC) resulted in a 100% reduction in microfilaremia on day 1 (Table 1). DEC 2, 4, or 6 mg/kg/day for 10, 14, and 21 days reduced MFD by 100% at an unknown nadir time point (Table 1). DEC 200 mg three times per day for 20 days had a reduction of 63%, and DEC 200 mg three times per day for 20 days followed by a monthly dose of 200 mg for 6 months had an 80% reduction, with time to nadir occurring at month 7 for both regimens (Table 1).

### Moxidectin

A recent trial assessed the efficacy of moxidectin (MOX) 2 mg given as a single dose on its potential to reduce *L. loa* microfilaremia. MOX was compared with standard IVM therapy and showed a slower and lower reduction in microfilaremia than IVM, reaching a nadir of about 50% at day 30 (Table 1) [36].

### Levamisole

Three Levamisole treatment groups of 1, 1.5, and 2.5 mg/kg/day were administered as a single dose and had a percent reduction in microfilaremia of −10%, 8%, and 13% at nadir, respectively (Table 1). The nadir was reached on day 2 with doses of 1.5 and 2.5 mg/kg/day, and on day 30 with a dose of 1 mg/kg/day (Table 1).

### Imatinib

Three Imatinib treatment groups of 200, 400, and 600 mg per day were administered for 1, 2, and 3 days, respectively (Table 1). Imatinib 400 mg/day for 2 days had a 40% reduction on day 4, 200 mg/day for 1 day had a 21% reduction on day 7, and 600 mg/day for 3 days had a 17% reduction on day 7 (Table 1).

### Combinations treatments

There were two IVM combination treatments with doxycycline and ALB. Doxycycline 200 mg per day for 6 weeks with a single dose of IVM 150–200 µg/kg/day at month 4 had a reduction of 100%, with nadir measured at 12 months (Table 1). ALB 400 mg twice daily for 21 days followed by a single dose of IVM 150 µg/kg/day on day 23, achieved a 96% nadir reduction at 1 month (Table 1).

### Other regimens: antimalarial drugs

One controlled clinical trial assessed the efficacy of chloroquine 600 mg/day, amodiaquine 800 mg/day, and artesunate 200 mg/day, each administered for 3 days, and quinine 600 mg twice per day for 5 days, for their activity against *L. loa* microfilaremia (Table 1). The observed reduction in microfilaremia by these antimalarial regimens was 51%, 22%, 14%, and 31%, respectively (Table 1). The time to nadir was 60 days for chloroquine and quinine and 90 days for amodiaquine and artesunate (Table 1).

### Clinical treatment outcomes

Of the 27 included studies, only three reported clinical outcomes both before treatment and during the follow-up period.

In the IVM study by Martin-Prevel et al., a clinical score for loiasis that included pruritus and Calabar swellings was presented [26]. A score of 0 was given for symptoms that were neither frequent nor intense, 1 for symptoms that were either frequent or intense, and 2 for symptoms that were both frequent and intense [26]. The loiasis clinical score was compared before treatment and at the study’s conclusion. Improvement was seen in 13 subjects, a decrease in 4, and no change in 13 [26]. Among participants receiving a single IVM dose of 300 µg/kg, 73% experienced symptoms before treatment compared with 33% after treatment, measured between 92 and 109 days post treatment [26]. Among participants receiving a single IVM dose of 400 µg/kg, the percentage experiencing symptoms remained unchanged at 40% [26]. However, the two patients who initially had a score of 2 reported a score of 1 during follow-up [26]. The authors found a correlation between the mean decrease in microfilaremia and clinical improvement, noting that the 13 subjects who showed clinical improvement also had a significantly greater reduction in *L. loa* microfilarial counts [26].

In the MBZ study by Hoegaerden et al., 40% of subjects receiving 100 mg three times per day for 45 days, 57% of those receiving 500 mg per day for 45 days, and 100% of those receiving 500 mg three times per day for 21 days had Calabar swellings before treatment [17]. After treatment, only one participant, who received 500 mg three times per day, experienced a recurrence of Calabar swelling 29 days after the treatment ended [17]. Among all participants in the various MBZ treatment groups, 63% experienced pruritus before treatment, which resolved in all participants during the 3–12-month follow-up period [17].

In Murgatroyd et al.’s study on loiasis treated with 2, 4, or 6 mg/kg of DEC for 10, 14, or 21 days, 12% of participants had worms under the conjunctiva and 100% had Calabar swellings before treatment [27]. All participants remained symptom free after treatment, with the exception of one who experienced a recurrence of Calabar swellings [27]. However, this was 3 months after re-entering an endemic area of the disease [27]. The authors suggested the possibility of reinfection in this case [27].

### Risk of bias of individual studies

**Fig. 6:**
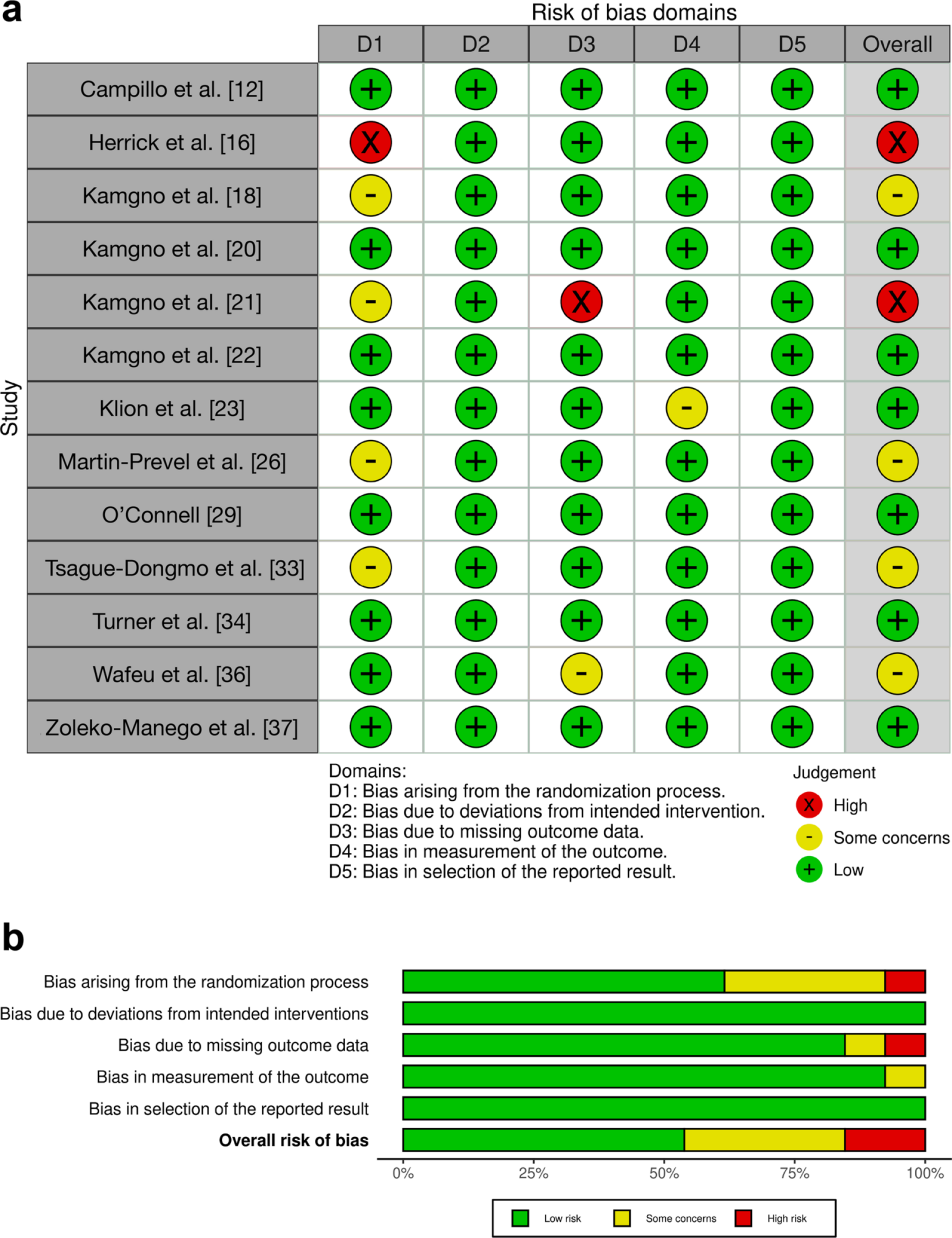
RoB2 analysis results: assessing bias in randomized trials. **a** Results of risk of bias assessments for included randomized trials. **b** Summary of risk of bias assessments for included randomized trials

**Fig. 7:**
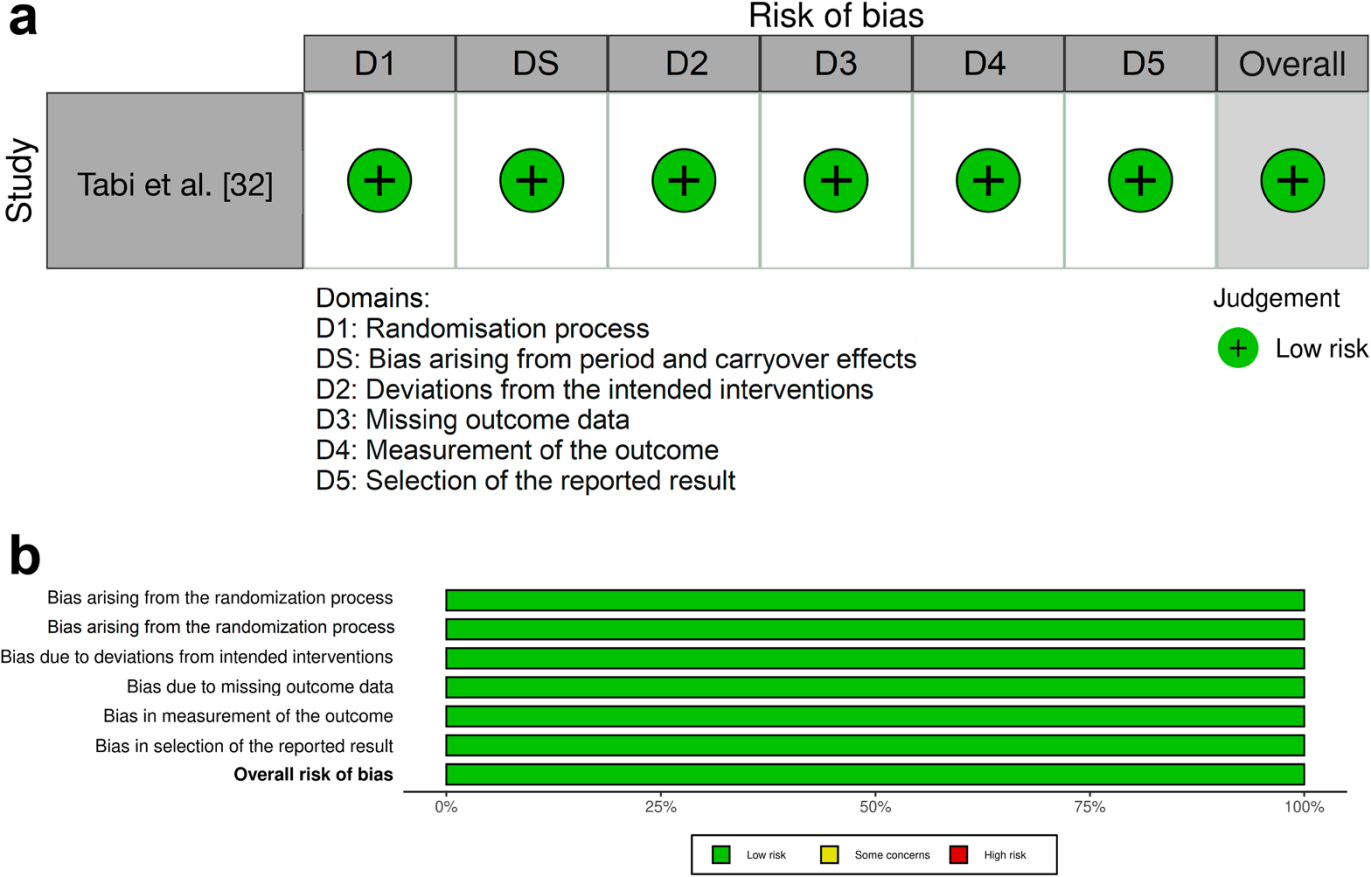
RoB2 crossover analysis results: assessing bias in randomized crossover trial. **a** Results of risk of bias assessments for included randomized crossover trial. **b** Summary of risk of bias assessments for included randomized crossover trial

**Fig. 8:**
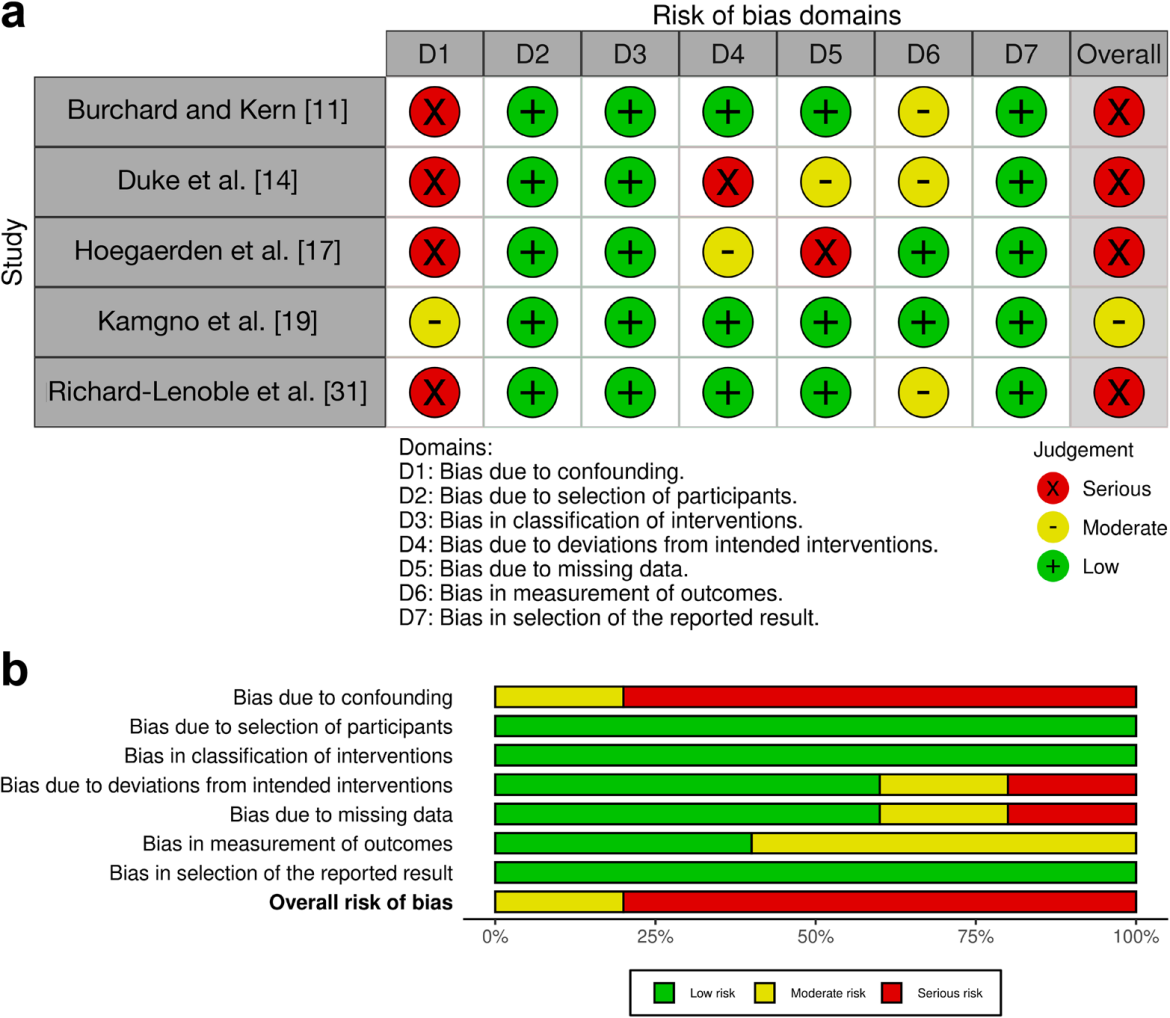
ROBINS-I analysis results: assessing bias in nonrandomized studies of interventions. **a** Results of risk of bias assessments for included nonrandomized studies of interventions. **b** Summary of risk of bias assessments for included nonrandomized studies

**Fig. 9:**
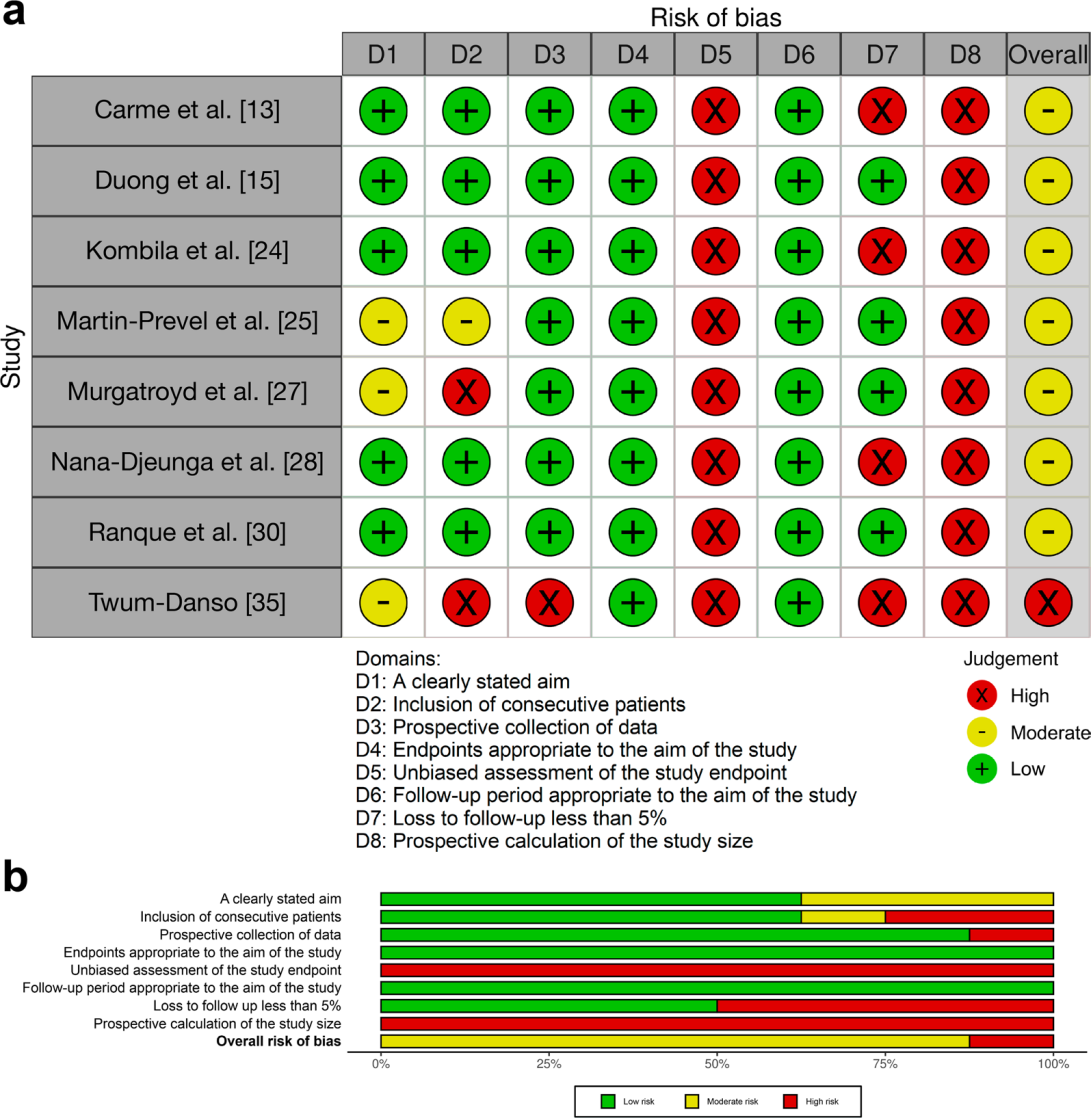
MINORS analysis results: assessing bias in noncomparative studies. **a** Results of risk of bias assessments for included noncomparative studies. A green plus sign ( +) indicates that the domain was reported and adequately addressed, earning 2 points; a yellow minus sign (−) indicates that the domain was reported but inadequately addressed, earning 1 point; and a red cross ( ×) indicates that the domain was not reported, earning 0 points [44, 45]. For noncomparative studies, the maximum possible MINORS score is 16. For this review, an overall high risk of bias is assigned to studies scoring 8 or less across all domains, a moderate risk of bias to those scoring between 9 and 14, and a low risk of bias to those scoring between 15 and 16 [46]. **b** Summary of risk of bias assessments for included noncomparative studies, illustrates the proportion of studies categorized as having low, moderate, or high risk of bias for each MINORS domain

Of the 27 studies included in the analysis, 8 studies received an overall low risk of bias judgment, 12 studies received a score indicating moderate or some concerns for bias, and 7 received a high or serious risk of bias categorization. Figures 6, 7, 8, and 9 present the results of our risk of bias assessments based on relevant outcomes, as well as the summary across all studies. Only randomized controlled clinical trials received a low risk of bias judgment, with the majority, 62% (8/13), being assessed as low risk and only 15% (2/13) assessed as high risk (Fig. 6). In addition, one included randomized crossover trial, assessed separately with an additional domain, received a low risk of bias judgement (Fig. 7). Among the nonrandomized studies comparing at least two study arms, the majority (4/5) were judged to have a high risk of bias (Fig. 8a). The quality of these studies was most frequently compromised by bias arising from confounding (Fig. 8b). For noncomparative studies, seven were rated as moderate risk and one as high risk (Fig. 9).

## Discussion

The aim of this systematic review was to evaluate the efficacy of various treatment regimens on the reduction of microfilaremia in patients with loiasis. The main findings indicate that rapid microfilaricidal drugs, IVM and DEC, significantly reduce microfilarial levels, while benzimidazole anthelmintic drugs, ALB and MBZ, show moderate-to-high efficacy depending on the dose and, foremost, the duration of treatment regimens. Higher doses and longer treatment durations of ALB resulted in greater reductions in microfilaremia, ranging from 80% to 98%, with nadir typically observed within 12 months of treatment. MBZ exhibited similar dose and duration dependence, achieving up to 100% reduction of microfilaremia generally within 60–200 days for the most efficacious regimens. IVM showed increased efficacy with higher treatment dosage in single dose regimens and a rapid onset of activity within 30 days after treatment. Repeated administration over prolonged periods was similarly associated with high efficacy, with reductions of microfilaremia up to 92% for doses between 200 and 400 µg/kg/day. DEC consistently demonstrated rapid and high efficacy, with certain regimens achieving a 100% reduction of microfilaremia.

### Albendazole and mebendazole

ALB demonstrated a wide range of treatment responses depending on both dosage and treatment duration. Short-term treatments of ALB at 400 mg/day, 400 mg twice per day, and 600 mg per day for 1–3 days resulted in modest microfilaremia reductions between 28% and 63% [19, 32, 33]. However, when administered at 200 mg or 400 mg twice daily over 21–35 days, ALB achieved much more substantial reductions, ranging from 80% to 98% [23, 37]. Moreover, ALB 800 mg bimonthly achieved a greater reduction in microfilaremia with extended treatment, yielding a 62% reduction when administered for 11 months, compared with a 32% reduction with the 3-month regimen [22]. These findings indicate that extended treatment durations, continuous administration, and higher doses of ALB maximize its efficacy in reducing microfilaremia. Similarly, MBZ showed no reduction of microfilaremia at a lower dose and shorter treatment length [19], compared with reductions of up to 100% with higher doses and extended treatment durations of 21–45 days [17]. This indicates that the efficacy of ALB and MBZ are likely dose and duration dependent.

### Ivermectin

The efficacy of IVM varied notably depending on the dose and frequency of administration. Among single doses of IVM, lower doses (5–10 µg/kg/day) resulted in minimal reductions (7–12%) [31], whereas higher doses (200–400 µg/kg/day) achieved substantial reductions between 88% and 92% [13, 15, 16, 25, 26, 31]. A single dose of 150–200 µg/kg achieved the most significant reduction of 98% [35]. Importantly, nadir was generally measured within 30 days of treatment initiation, highlighting the rapid onset of IVM’s activity on microfilaremia. Given that the time to nadir was measured at an early stage, it is unlikely that the observed reduction in microfilaremia was distorted by the emergence of a new generation of microfilariae, given the parasite’s life cycle and the fact that IVM lacks macrofilaricidal activity [4]. Administering 50–300 µg/kg/day for multiple days (2–7 days) [31] did not significantly improve microfilaremia reduction compared with giving the same dose as a single-day treatment, suggesting that the efficacy plateaus after the first day. However, repeated administration of single dose IVM at wider dosing intervals over extended periods of time showed remarkable efficacy, with monthly doses of 200 µg/kg for 6 months achieving a 99% reduction [24] and yearly doses for 18 years resulting in complete elimination of microfilaremia (100% reduction) [28]. These findings underscore IVM’s efficacy, particularly with higher single doses or prolonged dosing.

### Diethylcarbamazine

DEC was highly efficacious, with several regimens achieving a 100% reduction in microfilaremia [16, 27]. DEC doses of 2–8 mg/kg/day administered for 1–21 days resulted in complete microfilarial clearance at nadir [16, 27]. However, a 20-day regimen of 200 mg three times per day achieved only 63% reduction, which increased to 80% with an additional monthly dose over 6 months [14]. This demonstrates DEC’s high potential for microfilaremia reduction, especially with longer-term and higher-dose treatment regimens.

### Moxidectin

Similarly, one trial assessed the safety and efficacy of MOX to evaluate its potential use for onchocerciasis elimination in loiasis-endemic areas [36]. Given the risk of SAEs in individuals with high *L. loa* microfilaremia, a low dose of MOX was administered to participants with low MFD to prioritize safety [36]. The trial results showed no SAEs or grade 3 or 4 adverse events, and the decrease in *L. loa* microfilaremia was significantly slower in the MOX group compared with the IVM group [36]. This slower reduction suggests that MOX may offer, at least at this low dose regimen, a safer alternative to rapidly acting microfilaricidal drugs, which are more likely to be associated with SAEs [36]. Although an almost 50% reduction of microfilaremia was observed, further research and clinical trials assessing higher doses of MOX and involving patients with higher *L. loa* MFD are needed to confirm the efficacy and safety of MOX on *L. loa* microfilaremia.

### Levamisole and imatinib

Levamisole and imatinib were less efficacious compared with other treatments [12, 29]. Single doses of levamisole (1.5 and 2.5 mg/kg/day) showed minimal reductions of microfilaremia (8–13%), with the lowest dose (1 mg/kg/day) resulting in negative efficacy (−10%) [12]. Imatinib also demonstrated a low reduction, which ranged from 17% to 40%, suggesting the limited efficacy of these medications in reducing *L. loa* microfilaremia [29].

### Combination treatments

Treatments combining IVM with other drugs showed promising results. ALB combined with a single dose of IVM reached a 96% reduction [37]. However, only 22% of patients receiving this combination treatment had a microfilarial load below 100 mf/ml at the 6-month follow-up [37]. Contrary to this finding, a cohort of patients with imported loiasis in Italy showed an almost complete cure with an ALB–IVM-based regimen, where 94% (15/16) of patients had a negative microfilaremia within 6 months after treatment (Additional File 1: Supplementary Table S1) [38]. The reason for this difference in parasitological cure between the study in an endemic region and the observation in returning travelers may be explained by differences between endemic and nonendemic settings. In nonendemic Italy, where the patients were treated, there was no possibility of re-infection which likely contributed to higher cure rates, in contrast to the trial in Gabon, where there is a potential for ongoing loiasis transmission. Similarly, patients in Gabon may be infected with a higher number of worms of varying age, which may also add to an overall lower efficacy of the drug regimen.

Overall, combination treatments with ALB and IVM demonstrate high efficacy in reducing microfilaremia. ALB is known as a slow-acting drug against *L. loa* microfilaremia but appears to gradually kill adult worms and to inhibit the reproductive process [37]. In contrast, IVM rapidly reduces microfilaremia, though this quick action has been associated with severe and serious adverse drug reactions in individuals with a high microfilaremia (≥ 8000 mf/ml) [37]. Therefore, initiating ALB may, at least in theory, safely reduce microfilariae prior to administering the rapidly acting IVM [37].

The combination of doxycycline and IVM showed a 100% reduction of microfilaremia [34]. However, it is particularly important to note that according to Turner et al., the reduction in microfilarial load was primarily due to the IVM treatment, as doxycycline does not target *L. loa* microfilaremia effectively owing to the absence of *Wolbachia* symbionts in *L. loa* [34]. In this study, participants received an oral dose of IVM 150 µg/kg 4 months after the start of the 42-day doxycycline treatment [34]. This suggests that while doxycycline may not directly reduce *L. loa* microfilaremia, it can be safely used in combination with IVM for co-infections with onchocerciasis without increasing the risk of severe adverse events.

The results suggest that combination therapies may enhance the efficacy of microfilarial reduction and provide a safer treatment option for patients with filarial co-infections and hypermicrofilaremic individuals, a factor of particular relevance in the tropical rain forest zones of West and Central African countries where co-infection with onchocerciasis occurs. Moreover, combination regimens highlight key considerations for treatments of filarial diseases in co-endemic settings, where IVM cannot be safely administered owing to the potential of serious adverse events in individuals with high *L. loa* microfilarial counts. These findings underscore the importance of the choice of medication for ensuring both safety and efficacy, as different treatment regimens can vary widely in their impact on microfilarial reduction and patient tolerance.

### Other treatment regimens: antimalarial drugs

Onchocerciasis control programs based on mass IVM treatment face significant challenges owing to the co-endemicity of loiasis, as IVM can induce serious adverse events in individuals with high *L. loa* microfilaremia [21]. Although antimalarial drugs, such as amodiaquine, artesunate, chloroquine, and quinine, have demonstrated some efficacy against filarial species, their efficacy is limited. Among these, chloroquine achieved the highest reduction at 51%, whereas artesunate showed the lowest reduction at 14% [21]. These results suggest that antimalarial drugs are insufficient for efficaciously reducing microfilaremia in co-endemic regions to ensure the safety of mass IVM treatment.

### Excluded studies

Four studies from nonendemic settings were excluded from the primary analysis of this systematic review owing to insufficient data on the evolution of microfilaremia. However, these studies present important findings on the proportion of patients achieving parasitological cure, which should be considered when assessing the efficacy of treatments for *L. loa* microfilaremia.

A retrospective analysis of 167 cases of imported loiasis in France evaluated the treatment efficacy of IVM and DEC alone and as a combination treatment [39]. IVM, administered at 200 µg/kg for 1–6 courses, resulted in negative microfilaremia in 52% (59/113) of patients [39]. DEC, given as a progressive dosage with an initial dosage of 10–75 mg and a final dosage of 200–400 mg over a 21-day period, led to no patients without microfilaremia [39]. However, IVM for 1 day followed by progressive dosage of DEC for 21 days achieved negative microfilaremia in 76.9% (20/26) of patients [39].

Another retrospective analysis of imported loiasis in France, involving 47 cases, assessed the efficacy of IVM, DEC, and ALB administered individually and as combined treatment regimens (DEC–ALB, DEC–IVM, and IVM–ALB) [40]. Neither IVM nor ALB, alone or combined with each other, resulted in amicrofilaremia [40]. In contrast, DEC alone or in combination with IVM achieved negative microfilaremia [40]. DEC alone led to negative microfilaremia in 6% of patients (1/11), and the one patient who received DEC–IVM was amicrofilaremic after treatment [40].

A larger retrospective study conducted across 11 reference centers for tropical medicine in seven European countries (Belgium, Finland, France, Germany, Italy, Spain, and Switzerland) also evaluated the efficacy of IVM, DEC, and ALB, both individually and in combination [41]. ALB 200–400 mg twice daily for 21–28 days followed by a single dose of 150–200 µg/kg/day of IVM resulted in the highest proportion of patients with negative microfilaremia, with 67% (14/21) achieving parasitological cure [41]. DEC, administered at 6 mg/kg/day for 21 days, was also efficacious, with 50% (37/74) of patients becoming amicrofilaremic [41]. In addition, DEC combined with IVM or ALB resulted in negative microfilaremia in 44% (7/16) and 38% (3/8) of patients, respectively [41]. IVM alone achieved negative microfilaremia in 18% (7/39) of patients, while ALB alone showed no success in completely clearing microfilaremia [41].

Finally, a study on imported loiasis in Italy described the outcomes of 16 cases that were treated with ALB 400 mg twice per day for 28 days followed by a single dose of 200 µg/kg/day of IVM [38]. Within 6 months after treatment, 94% (15/16) of patients had negative microfilaremia [38].

The data from these excluded studies, which are presented in Additional File 1: Supplementary Table S1, highlight important results on the efficacy of various treatment regimens for *L. loa* microfilaremia, particularly combination treatments. Regimens that included DEC or IVM achieved higher rates of parasitological cure, while ALB alone was less effective. These findings underscore the importance of considering combination treatments for loiasis management.

### Strengths and limitations

The systematic evaluation of all available evidence for the assessment of the efficacy of antifilarial drugs on microfilaremia as performed in this analysis is so far unique. To date, no systematic review has compared all available treatment regimens for loiasis. A significant prior contribution to this field is the systematic review by Pion et al. from 2019, which focused on the effect of a single standard dose (150–200 μg/kg) of IVM on *L. loa* microfilaremia [42]. Our review extends beyond this by systematically comparing a broader range of treatments, thus providing information for a comparative evaluation of available therapeutic options.

Our review adhered to a registered protocol (PROSPERO CRD42024558036) and employed a comprehensive search strategy to capture relevant studies. The study selection process was strengthened by using multiple independent screeners, with double blinding during screening to reduce potential bias. In addition, we included both randomized and nonrandomized studies, with the use of multiple risk-of-bias tools (RoB2, ROBINS-I, and MINORS), allowing for a thorough assessment.

Despite the inclusion of multiple studies in the review, the overall quality of evidence remains limited, as most studies included in the analysis were rated as having a moderate (12) or high (7) risk of bias, with only a few considered to be of high quality (8). Furthermore, while some important treatment studies were identified, they had to be excluded as they did not meet the inclusion criteria or lacked usable data on microfilaremia during the follow-up period, despite their relevance. In addition, the lack of data on clinical treatment outcomes in many studies limits our ability to assess the broader impact of treatments beyond microfilaremia reduction.

### Future research priorities

Carefully tailored treatment protocols are essential to maximize efficacy and minimize adverse effects. Future research should focus on standardizing these protocols and exploring long-term effects to improve the management of loiasis infections. Moreover, studies should include patient-reported outcomes to better understand the impact of treatments from the patient’s perspective. Furthermore, research on occult loiasis, characterized by the absence of microfilariae in the blood but with clinical signs such as Calabar swelling or eye worm migration, is crucial [43]. Understanding occult loiasis will help determine the appropriate therapy and medication for those presenting with such symptoms, ensuring comprehensive and individualized patient care. These steps are vital for advancing the treatment of loiasis infections and improving patient outcomes.

## Conclusions

This systematic review reveals that the efficacy of therapeutic regimens is dose- and duration dependent for some anthelmintic drugs. Increasing dosage and treatment length improves the efficacy of ALB and MBZ. Similarly, higher single IVM doses of 200–400 µg/kg are more efficacious in reducing microfilaremia than low-dose regimens. Both IVM and DEC are favorable treatment choices due to their rapid and high activity against *L. loa* microfilaremia but pose considerable safety challenges and concerns for individuals harboring a high microfilarial load.

## Supplementary Information

Additional file 1: Table S1. Important non-endemic studies excluded from primary analysis due to insufficient data.

## Data Availability

All data are available within the paper and its Supplementary Information.

## Abbreviations

ALB: Albendazole
DEC: Diethylcarbamazine
IVM: Ivermectin
*L. loa*: *Loa loa*
MBZ: Mebendazole
MFD: Microfilarial density
MINORS: Methodological index for nonrandomized studies
MOX: Moxidectin
RoB2: Risk of bias in randomized trials
ROBINS-I: Risk of bias in nonrandomized studies of interventions
SAEs: Serious adverse events
