# Supplementary material for: Activity of antifilarial drugs on microfilaremia in the treatment of loiasis: a systematic review
Source: Parasit Vectors. 2025 Dec 10;19:30. doi: 10.1186/s13071-025-07189-w (PMC12801498; doi:10.1186/s13071-025-07189-w)
Supplement: Supplementary file 1 — Additional file 1: Table S1. Important non-endemic studies excluded from primary analysis due to insufficient data. [file 13071_2025_7189_MOESM1_ESM.docx]

Additional file 1: Table S1 Important non-endemic studies excluded from primary analysis due to insufficient data

| Author, Date [Reference] | Country | Treatment | Dose | Treatment Length | Negative Mf |
| --- | --- | --- | --- | --- | --- |
| Bouchaud et al., 2021 [39] | France | IVM | 200 µg/kg per course | 1-6 courses | 59/113 (52.2%)^a^ |
|  |  | DEC | progressive dosage: initial dosage 10-75mg, final dosage 200–400 mg | 21 days | 0/10 (0%)^a^ |
|  |  | IVM then DEC | 200 µg/kg IVM per course + progressive DEC dosage (initial dosage 10-75 mg, final dosage 200–400 mg) | 1 day IVM + 21 days DEC | 20/26 (76.9%)^a^ |
| Gantois et al., 2013 [40] | France | IVM | NA | NA | 0/27 (0%)^b^ |
|  |  | DEC | NA | NA | 1/11 (6%)^b^ |
|  |  | ALB | NA | NA | 0/4 (0%)^b^ |
|  |  | DEC-ALB | NA | NA | NA^b^ |
|  |  | DEC-IVM | NA | NA | 1/1 (100%)^b^ |
|  |  | IVM-ALB | NA | NA | 0/2 (0%)^b^ |
| Gobbi et al., 2018 [41] | Belgium, Finland, France, Germany, Italy, Spain, & Switzerland | IVM | 150-200 µg/kg/day | 1 day | 7/39 (17.9%)^c^ |
|  |  | DEC | 6 mg/kg/day | 21 days | 37/74 (50.0%)^c^ |
|  |  | ALB | 200-400 mg BID | 21-28 days | 0/5 (0%)^c^ |
|  |  | DEC+ALB | 200-400 mg BID ALB + 6 mg/kg/day | 21-28 days ALB + 21 days DEC | 3/8 (37.5%)^c^ |
|  |  | DEC+IVM | 150-200 µg/kg/day IVM + 6 mg/kg/day DEC | 1 day IVM + 21 days DEC | 7/16 (43.7%)^c^ |
|  |  | ALB+IVM | 200-400 mg BID ALB + 150-200 µg/kg/day IVM | 21-28 days ALB + 1 day IVM | 14/21 (66.7%)^c^ |
| Gobbi et al., 2019 [38] | Italy | ALB+IVM | 400 mg BID ALB + 200 µg/kg/day IVM | 28 days ALB + 1 day IVM | 15/16 (93.8%)^d^ |

^a^disapperance of clinical symptoms and negative microfilaremia

^b^disappearance of microfilaremia

^c^negative microfilaremia and normal eosinophil count

^d^negative microfilaremia

Note: The references were listed in the Reference section in the main text.

Abbreviations: ALB, albendazole; BID, twice a day; DEC, diethylcarbamazine; IVM, ivermectin; Mf, microfilaremia
